# *Overcome the Fear (Vencer el Miedo)*: using entertainment education to impact adolescent sexual and reproductive health and parent-child communication in Mexico

**DOI:** 10.1186/s12889-022-14853-8

**Published:** 2022-12-16

**Authors:** Jorge A. Montoya, Aaron Plant, Deborah Neffa-Creech, Cecilia Orvañanos, Kriss Barker

**Affiliations:** 1 Sentient Research, 231 North Walnuthaven Drive, West Covina, CA 91790 USA; 2 Population Media Center, Recta a Cholula 1611-12, Cholula, 72760 Puebla, Mexico; 3 Population Media Center, 30 Kimball Ave., Suite 302, South Burlington, VT 05403 USA

**Keywords:** Entertainment-education, Telenovela, Adolescent health, Sexual and reproductive health, Family planning, Surveys, Evaluation, Mexico

## Abstract

**Background:**

Adolescents in Mexico experience high pregnancy and birth rates. A collaboration with Grupo Televisa led to the development of an entertainment-education *telenovela* intervention, *Overcome the Fear (OTF)*, which aired in 2020 to a national audience and addressed adolescent sexual and reproductive health (SRH) topics. This study details the development and evaluation of *OTF*’s impact on adolescent contraceptive practices and parent-adolescent SRH communication in Mexico.

**Methods:**

We conducted cross-sectional survey interviews (street-intercept and telephone) with 12–19-year-olds (*n* = 1640) and parents of adolescent children (*n* = 820) post-broadcast across Mexico’s five most-populated metropolitan zones. Quotas were implemented for gender, zone, and *OTF* viewership (viewer vs. non-viewer). Bivariate statistics and multivariable binary logistic regression models assessed the relationship between *OTF* viewership (including parent-adolescent co-viewing) and adolescent contraceptive practices and parent-adolescent SRH communication. Adolescent and parent data are not dyadic and were analyzed separately.

**Results:**

Nearly half of adolescents (47.9%) and parents (47.7%) were viewers. Among adolescents, bivariate analyses suggest that viewers had less negative attitudes towards contraception (*p* < .001). Logistic regression models suggest that adolescent viewers were more likely to seek out information about contraception (*p* < .001) and unhealthy romantic relationships (*p* = .019), and to use contraception other than condoms (*p* = .027) and dual contraception (*p* = .042) in the last 3 months. Among parents, bivariate analyses suggest that non-viewers had more positive attitudes towards abstinence (*p* = .045) and more negative attitudes towards contraception and communication with adolescents about sex (*p* = .001). Logistic regression models suggest that parent viewers were more likely to have talked with adolescent children about sexual relations (*p* < .001), contraceptive methods (*p* = .01), condoms (*p* = .002), and abstinence (*p* = .002) in the last 3 months. Parent-adolescent co-viewing of *OTF* was also significantly related to certain outcomes in bivariate analyses.

**Conclusions:**

This study suggests that viewership of a high-quality entertainment-education *telenovela* informed by extensive formative research is related to adolescent health outcomes and to parent-adolescent SRH communication on a country-wide scale in Mexico. Entertainment-education remains an underutilized public health strategy, despite its promise to engage viewers and motivate healthful behaviors.

**Supplementary Information:**

The online version contains supplementary material available at 10.1186/s12889-022-14853-8.

## Background

Adolescents (ages 12 through 19 years) in Mexico experience high birth rates, accounting for 17% of total births in 2019 [1]. While 9 in 10 Mexican adolescents are aware of at least one contraceptive method, among those with sexual onset (21.2%), 15.8% of males and 28.5% of females reported not using any contraceptive method at last intercourse in the country’s 2018–19 National Health and Nutrition Survey [2]. Among adolescents who had initiated sex, 79.2% of males and 54.9% of females reported using the male condom at last intercourse [2]. Further, nearly half (46.2%) of adolescent females who had initiated sex reported being pregnant at least once [2].

Entertainment-education (EE), also referred to as edutainment, is an intervention approach that has shown promise at improving sexual and reproductive health (SRH) outcomes among adolescents and young adults, including knowledge, behaviors (e.g., condom use), and the testing and management of sexually transmitted infections [3–5]. EE capitalizes on the entertainment value of interventions by engaging audiences in the storylines of characters who deliver health messages and model healthy behaviors [6]. In the United States, several EE television series or soap operas have focused on youth SRH and family planning [7–9]. EE *telenovelas* (Spanish-language soap operas with a finite number of episodes) have been used for decades in Mexico in attempts to address a wide range of health and social issues (e.g., literacy, adult-education, gender equality, poverty, and violence), as well as SRH [6, 10–12].

In addition to reaching primary target audience members (e.g., adolescents) with SRH messages, EE *telenovelas* offer the opportunity to reach other important target audiences, such as parents, who can influence adolescent SRH decision-making. Studies have demonstrated the impact of parent-child communication on adolescent SRH outcomes, including abstinence, sexual initiation, and condom use [13–17]. In Mexico, several studies found that parent-child communication about sexual risks before sexual initiation was associated with adolescents’ use of contraceptive methods, including condoms [18–21].

*Telenovelas* focused on SRH can offer opportunities for parent-child communication about these topics, particularly when parents and adolescents watch it together, or co-view [22]. Parent-adolescent co-viewing has been associated with a greater sense of connectivity [23], discussions about the television content [24], and SRH communication [25]. Further, television co-viewing can impact adolescent beliefs and behaviors, in part due to the adolescents’ belief that parental co-viewing is an endorsement of the content [26]. Studies suggest that parent-adolescent co-viewing can be linked to delayed sexual initiation and lower “positive sex expectancies” (e.g., sex can make one feel more grown up, attractive, loved, and wanted) [24, 27].

This study investigates the impact of a recent EE *telenovela* intervention on adolescent contraceptive practices and parent-adolescent communication about SRH topics in Mexico, as well as the relationship between parent-adolescent co-viewing and these outcomes. We describe the development of a 47-episode *telenovela, Overcome the Fear (Vencer el Miedo*), that aired nationwide in 2020, followed by an evaluation using two large-scale, cross-sectional surveys of adolescents and parents of adolescents.

### Theoretical background for entertainment-education telenovelas

EE in Mexico originated with Miguel Sabido’s use of the methodology in *telenovelas* in the late 1960s and early 1970s, a model that has since been disseminated throughout the world [6]. In Sabido’s EE approach, characters encounter challenging situations and relational tensions that require them to make decisions yielding positive or negative consequences. Specifically, a *transitional* character, designed to resonate with the target audience, ultimately enacts a desired behavior (e.g., an adolescent uses a condom or a parent talks with their adolescent child about sex), but only after wavering between decisions to enact or forgo the desired behavior. The build-up to these decisions begins with an *incident* where the transitional character encounters a difficult situation in which they must make a choice. This is followed by positive and negative characters attempting to *influence* the decision. Shortly after, the transitional character makes a *decision,* which is followed by *consequences* – negative consequences (e.g., getting a sexually transmitted infection (STI) or watching their parent-adolescent relationship grow distant) when the desired behavior is not chosen, or positive consequences (e.g., peace of mind practicing safer sex or developing a trusting relationship with their adolescent child) when the desired behavior is enacted. This cycle is repeated as the transitional character’s storyline unfolds. Ultimately, the tensions influencing the character’s decisions are resolved and the character is transformed as they make positive choices and reap the rewards of their healthy behaviors.

Sabido’s EE approach is grounded in two key theoretical frameworks – social cognitive theory [28, 29] and the extended elaboration likelihood model [30]. Social cognitive theory posits that people can learn behaviors by observing and imitating behaviors modeled by others. However, the extent to which an observer is motivated to enact the modeled behavior is influenced by *identification*, or perceived similarity with the actor of the behavior (i.e., homophily); *self-efficacy*, or the belief that one has the ability to perform the modeled behavior; and *outcome expectancy*, or the perceived consequences (negative or positive) resulting from enacting the behavior. In the context of SRH, seeing similar others successfully engage in healthy behaviors can increase the observer’s belief that they have the ability to enact the same behavior and can expect the same positive consequences or rewards for engaging in that behavior.

The extended elaboration likelihood model emphasizes the importance of *transportation*, a mental process where viewers are absorbed in the dramatic elements of the entertainment program and are less likely to resist or counterargue the embedded persuasive messages [30]. Green and Brock described this process as “an integrative melding of attention, imagery, and feelings” into the narrative [31]. Hence, viewers, listeners, or readers may become immersed in the story such that they are *transported* from their immediate surroundings into another reality where they become highly involved with characters and their situations [31]. Slater and Rouner argue that transportation is necessarily mediated by storyline appeal, production quality, and the unobtrusiveness of the persuasive message [30]. Therefore, an EE series with high-production value that seamlessly weaves in the persuasive subtext is more likely to influence the beliefs, attitudes, and behaviors of the target audience.

### Developing the *Overcome the Fear* telenovela

Using this theoretical framework, Population Media Center (PMC) and Grupo Televisa, the largest television network in Mexico, co-produced *Overcome the Fear (OTF)*, a 47-episode *telenovela* that incorporates transitional characters in tailored storylines. These characters were designed to model behaviors for parents and adolescents to address unplanned pregnancies among youth in Mexico. Grupo Televisa’s long-standing and successful track record of producing high-quality *telenovelas*, promoted across multiple media channels before and during broadcast, ensured a broad audience base would be reached, engaged, and sustained.

The development of *OTF* began with secondary research in 2015 examining trends of unplanned pregnancies and family planning practices in Mexico among adolescents and young adults. Among other vital statistics, the research revealed that birth rates among women 19 years old and younger increased between 2000 and 2014 while birth rates among older women decreased during the same time period [32, 33]. Additionally, a literature review indicated that parents can play an integral role in SRH education and related risk behaviors among their adolescent children in Mexico. For instance, adolescents whom rate their communication with parents as good are more likely to report fewer sexual partners [34], and parent-adolescent discussions about sexual behaviors are more likely to lead to condom use at first sex [18]. In Mexico, parent-adolescent discussions about SRH (e.g., condom use) can be rare, with discussions primarily focused on abstinence [17]. Further, parent-adolescent communication about sex can vary based on adolescents’ gender and sexual experience, with males and sexually active adolescents more likely to report such communication [35]. Regardless of socioeconomic status (SES), parents in Mexico possess greater knowledge about SRH compared to their adolescent child, as well as greater comfort levels with communication [36] – this suggests that focusing on parent-child communication could help to improve SRH outcomes for their adolescent children. Based on the findings from this secondary research, two target segments for the EE messaging were identified: adolescents (ages 12 through 19) and parents of adolescents in urban zones, where approximately 80% of Mexico’s population resides [37].

The secondary research was followed by qualitative research that included a series of key informant interviews and focus groups. Eleven interviews were conducted with representatives from multiple government and non-governmental organizations (NGOs) providing reproductive health services and/or working on reproductive health issues in Mexico. Key informant interviews were used to explore and understand the familial, political, legal, educational, and cultural contexts around SRH for adolescents; to identify community advisory group members; and to identify key partnerships with resources and services congruent with the *telenovela*’s EE outcomes.

Eighteen focus groups (total *n* = 155) were also conducted across two metropolitan zones (Mexico City and Guadalajara) and a rural area near Puebla with adolescents (15–19 years old) and young adults (20–24 years old). These groups were used to explore and understand youth’s: norms around romantic relationships, sexuality, and communication about sex with parents, other family members, and friends; daily routines, habits, and future aspirations; attitudes and beliefs around unplanned pregnancy and use of contraception; perceived barriers and facilitators to contraceptive use; and attributes of heroes and villains in their lives. This last area of discussion in the focus groups was used to inform the development of negative and positive characters in the *telenovela*.

Additionally, 30 focus group participants elected to participate in a WhatsApp photo exercise, in which they were asked to send in photos with descriptive captions of their home environment, school, work, neighborhood, friends, and something that is important to them. These photos were printed onto poster-sized card stock with captions and displayed during a workshop attended by the production team (described below) where members were asked to view the photographs, read the captions, and discuss their observations. In addition, 38 focus group participants elected to participate in a Facebook exercise, in which they were asked to “friend” a profile page set up for the study. This allowed the production team to review participants’ profiles, including posts of photos, hobbies, interests, and activities while gaining a detailed understanding of how adolescents dress and express themselves, as well as the look and feel of their homes and neighborhoods. Results from the WhatsApp and Facebook exercises were used to inform the development of realistic characters, wardrobe, situations, dialogue, set designs, and settings that the target audience would identify with.

Results from the formative research were incorporated into a three-day EE workshop with the *OTF* production team, which included the executive producer, director, set designers, location scouts, and script writers. A technical advisory group was also established to check the accuracy of medical and reproductive health information in the scripts and provide local resources for SRH services. The technical advisory group included government agencies and non-government organizations; UNFPA (United Nations Population Fund), CONAPO (*Consejo Nacional de Población*, or National Population Council), MexFam (*Fundación Mexicana para la Planeación Familiar*, or Mexican Foundation for Family Planning), Fundación Televisa, *Católicas por el Derecho a Decidir* (or, Catholics for the Right to Decide), INMUJERES (*Instituto Nacional de las Mujeres*, or National Institute of Women), SEDESA (*Secretaría de Salud*, or Secretary of Health), and SECTEI (*Secretaría de Educación, Ciencia, Tecnología e Innovación*, or Secretary of Education, Technology and Innovation). The *OTF* scripts were reviewed by EE experts at PMC who provided the writers with feedback and guidance on characters and their storylines.

A key partnership was formed with *MexFam,* an NGO in Mexico that delivers reproductive health services through a network of 85 service points, including 9 permanent clinics and 63 mobile clinic sites. *MexFam* also operates the *OrientaSex* call center with professional counselors to answer questions about SRH and provide referrals to clinics or other services. At the conclusion of each episode, a 20-second epilogue was delivered by cast members with a message about SRH followed by the *OrientaSex* hotline telephone number.

### Main characters and storylines in *OTF*

Two principal transitional characters, Marcela Durán and Lorenzo Bracho, and their storylines were designed to model the use of dual contraception among youth and parent-adolescent communication about SRH. Marcela (age 15 in the beginning of the series) is the adolescent heroine of the series and is manipulated by her boyfriend, Rommel, into committing a crime that lands her in juvenile detention for 3 years. Rommel does not visit her while she is in detention, but when she gets out, he tells her that he loves her and wants to have sex with her. It is clear, however, that Rommel is only interested in Marcela (now age 18) because she confesses to him that she is a virgin. Eventually they have sex but do not use a condom or other contraceptive method as Rommel convinces her that she can trust him. Some days later, Marcela misses her menstrual cycle, which leads her to think that she is pregnant. When she tells Rommel, he is angry with her and argues that she did not take care of herself. Marcela decides to go to a clinic where she finds out that she is not pregnant but has chlamydia, a curable STI. In one scene, the doctor recommends that she use a condom along with another form of contraception (dual contraception) to prevent STIs and unplanned pregnancy. Ultimately, she realizes that she is in an unhealthy relationship with Rommel and decides to leave him and take better care of herself.

Lorenzo is Marcela’s older brother and a single father to a daughter, Areli (age 10 in the beginning of the series). At age 13, Areli becomes a teenager and has questions about sex; however, her father, Lorenzo, does not know how to talk to her about SRH issues. Areli and her best friend decide to search for answers online but accidentally visit a pornographic website. This gives Elvira, Lorenzo’s fiancée and soon-to-be stepmother to Areli, the opportunity to manipulate Lorenzo into punishing Areli for looking up information on her own. As Lorenzo continues to struggle with how to talk with Areli about sex, Elvira fuels his fears about his daughter becoming a young woman, and he eventually decides to prohibit her from having a boyfriend or leaving the house, other than to go to school. This causes Areli to distance herself from her father, and she does not get the support or information she needs. In the end, Lorenzo realizes his mistake and repairs his relationship and communication with his daughter. He also breaks up with Elvira. Ultimately, with Lorenzo’s support, Areli goes to a clinic to get information about contraceptives.

### *OTF* television and online reach

*OTF* aired January 20 through March 22, 2020, on Grupo Televisa’s *Canal de las Estrellas* (*Channel of the Stars*) on weeknights (6:30–7:30 pm), reaching on average 3.5 million nightly viewers. In addition, multiple transmedia components (e.g., social media accounts and *OTF* website) were used to amplify the main messages across multiple media platforms. These components garnered 415,000 interactions (e.g., social media likes and comments), 20 million video views, and a total reach of 42 million individuals across Facebook, Instagram, YouTube, and Twitter. In addition, Grupo Televisa’s main web page for *OTF* (on LasEstrellas.tv) reached 510,000 users with 2.6 million page views, 1.4 million episode views, and 225,000 video clip views, averaging 66,000 views every time an episode aired.

## Methods

Survey interviews with adolescents (*n* = 1640) and parents of adolescent children (*n* = 820) were conducted post-broadcast (series finale air date was March 22, 2020) to assess the potential impact of *OTF* on adolescents’ contraceptive use behaviors and parent-adolescent SRH communication. Cross-sectional street-intercept (25.5%) and telephone (74.5%) interviews took place from March 25 through May 8, 2020, across the five most-populated metropolitan zones in Mexico: Mexico City (and its surrounding suburban areas), Guadalajara, Monterrey, Puebla, and Tijuana. Originally, all interviews were going to be conducted via street-intercept, but due to safety concerns related to the COVID-19 pandemic, remaining surveys were collected by telephone.

To be included in the study, participants had to have at least one functioning television at home and either view broadcast television anywhere from 6:00 pm to 10:00 pm a minimum of three evenings from Monday through Friday or have viewed a drama series (or *telenovela*) through an online platform. Adolescents were eligible to participate if they were 12–19 years old, were not expecting children, and were not parents. Parents were eligible if they were 30–50 years old and had at least one 12–19-year-old child at home. Parent and adolescent participants were not recruited as dyads, so there was no known relationship between parent and adolescent participants interviewed. Quotas were implemented for gender, metropolitan zone of residence, and viewership of *OTF* (viewer vs. non-viewer). Interviews were conducted in Spanish language by trained, local interviewers. Interviews had an average duration of 25 minutes (median 21 minutes).

For street-intercept interviews, potential participants were approached at multiple locations within each city with high foot traffic (e.g., parks, malls, shopping centers, and schools or colleges). For telephone interviews, potential participants were identified through an automated dialing process targeting residents from the five metropolitan zones. Once potential participants were identified, they were read a brief introduction and informed consent information. Parental consent was obtained before seeking assent from minor adolescent participants (younger than 18 years old). Interviews were conducted with those who agreed to participate. The refusal rate was 25.9% among those who received the introduction and informed consent information. No incentive was offered for participation. This study was reviewed and approved by the Western Institutional Review Board.

### Measures

Survey instruments measured *OTF* viewing behaviors (including co-viewing with others), parental communication with adolescent children about SRH topics (including unhealthy relationships), and adolescent contraceptive practices and related information-seeking behaviors.

### Demographics and background characteristics

Multiple demographic and background characteristics were obtained including age, gender, SES, metropolitan zone of residence, parental status (mother, father, or guardian), number and gender of adolescent children at home, romantic relationship status, employment status, and highest level of education attained. SES was assessed using six questions from the *Asociación Mexicana de Agencias de Inteligencia* (Mexican Association of Intelligence Agencies) [38].

#### OTF viewing

Respondents were asked if they had viewed *OTF*, followed by the number of episodes typically viewed per week. Respondents who viewed two or more episodes per week were classified as “viewers” (*n* = 391 parents; *n* = 786 adolescents) and those who did not view any episodes, or viewed one or fewer episodes per week, were classified as “non-viewers” (*n* = 429 parents; *n* = 854 adolescents). Among non-viewers, only 9 parents and 14 adolescents viewed one or fewer episodes per week – the rest did not view any episodes. We considered it highly unlikely for those who viewed one or fewer episodes per week (of the five that aired weekly) to understand Lorenzo’s or Marcela’s storylines and decision-making processes from *incident* to *consequence* where characters were modeling behaviors.

#### Parent-adolescent co-viewing

*OTF* viewers were also asked whether they would typically watch episodes with others or alone. Those who watched with others were asked to specify with whom, and the interviewer selected all who were mentioned from a list of options, which included son(s), daughter(s), mother, and father.

#### Communication with adolescent child about SRH and relationships (parents)

We asked parents whether (yes/no) they had talked to their adolescent children in the last 3 months about sex, contraceptive methods, condoms, abstinence, and unhealthy romantic relationships.

#### Sexual practices and contraceptive use (adolescents)

We asked adolescents about sexual onset (whether they have ever had vaginal sex), condom use at last sex, and frequency of the following in the past 3 months: vaginal sex, condom use, use of other contraceptive methods, and use of dual contraception.

#### Attitudes toward talking to adolescent children about SRH (parents)

Parents’ attitudes were assessed with eight agreement statements using a 7-point Likert scale (1 = completely disagree, 7 = completely agree). We explored positive and negative attitudes related to communication with adolescent children about contraception and sex, but we could only ask a few questions for each topic due to time limitations. Three factors emerged using a principal components analysis extraction method with a Varimax rotation. Composite scores were computed for each factor keeping only items with loadings ≥ .30. The first factor, positive attitude toward talking to adolescents about sex and contraception (α = .71, *M* = 19.28, *SD* ± 3.14), included three statements: *It is a parent’s responsibility to talk to their adolescent children about sex*; *It is important for parents to make sure their adolescent children know about contraceptive methods*, and *When parents talk to their adolescent children about contraception, it will help keep them from getting into trouble later.* The second factor, positive attitude towards abstinence (Spearman-Brown reliability coefficient = .51; *M* = 8.70, *SD* ± 3.65), included two statements: *Adolescent children should abstain from sex until they are adults*, and *Having sexual intercourse is something only married people should do.* The third factor, negative attitude towards contraception and talking to adolescents about sex (Spearman-Brown reliability coefficient = .51, *M* = 6.21, *SD* ± 3.50), included two statements: *Talking to your adolescent children about sex will compel them to have sex;* and *Getting contraception for your adolescent child will push them into having sex.* Due to the limited number of questions and items per factor, these are acceptable reliability values [39].

#### Attitudes about sexual practices and contraception use (adolescents)

Adolescent attitudes were assessed with 14 agreement statements on a 7-point Likert scale (1 = completely disagree, 7 = completely agree). Four factors emerged using a principal components analysis extraction method with a Varimax rotation. Composite scores were computed for each factor keeping only items with loadings ≥ .30. The first factor, negative attitude towards contraception use (α = .65, *M* = 14.03, *SD* ± 7.0), included five statements: *A girl who has contraceptives is probably having sex with different guys; A guy who carries condoms with him is just looking for sex; Using a condom means you’re not really faithful to the person you’re having sex with; You only need to use condoms if the person you are having sex with has had more than one partner;* and *You don’t have to use condoms during sex if you are only with one partner*. The second factor, positive attitude towards contraceptive use (Spearman-Brown reliability coefficient = .55, *M* = 12.21, *SD* ± 2.74), included two statements: *A girl who uses contraceptives is responsible*, and *It’s smart for a girl to have condoms with her if she is sexually active*. The third factor, positive attitude towards abstinence (α = .66, *M* = 13.33, *SD* ± 6.28), included four statements: *Having sex as an adolescent is a form of disobedience; People should abstain from having sex until they are adults; Having sex when you’re an adolescent brings shame to a young woman and her family*; and *Having sex is something only married people should do*. The fourth factor, positive attitude towards premarital sex (α = .59, *M* = 11.90, *SD* ± 4.96), included three statements: *It’s okay for a girl and a guy to have sex even if they don’t plan on getting married; It’s okay for a girl to have sex with a guy who is not her steady boyfriend;* and *If you really love your boyfriend/girlfriend, you can have sex.* These are acceptable reliability values given the limited number of questions and items per factor [39].

### Analyses

We used descriptive statistics to characterize the samples and bivariate statistics (e.g., logistic regression, *t*-tests, and Chi-square tests) to assess the relationship between *OTF* viewership and demographic characteristics and attitudinal measures. We also used bivariate statistics to assess the relationship between parent-adolescent *OTF* co-viewing and outcomes of interest. Five multivariable binary logistic regression models assessed the relationship between *OTF* viewership and parental communication with an adolescent child about SRH and unhealthy romantic relationships in the last 3 months. Five multivariable binary logistic regression models also assessed the relationship between *OTF* viewership and adolescent sexual practices and contraceptive use. We used IBM SPSS (v 26) to perform all analyses with a 95% confidence interval (two-tailed).

## Results

### Parents

For the parent sample (*n* = 820), 50% were female, the average age was 39.7 years, and there was an even distribution by residential zone in Mexico. SES ranged from D (lowest) to C+ (highest) with a plurality in the middle C class (24.1%). A majority were married (57.1%) or cohabitating with their partner (17.1%). Most (72.9%) had only one adolescent child at home, and about half (51%) had only male adolescent children, compared to only female or both. The average age of the youngest adolescent child was 14.3 years (see Table 1).

**Table 1:** Demographic characteristics of parent sample by viewership of *Overcome the Fear*

	Overall (*n* = 820)	Viewer (*n* = 391)	Non-viewer (*n* = 429)	Bivariate estimate of viewership
	% *n* or *M* (SD)			OR (95% CI)
**Age**	39.7 (5.9)	39.6 (5.8)	39.7 (6.0)	1.00 (0.97, 1.02)
**Gender**				
Male	50.0	50.1	49.9	1 (Ref)
Female	50.0	49.9	50.1	0.99 (0.75, 1.30)
**Metro Zone**				
Mexico Valley	20.0	20.5	19.6	1 (Ref)
Guadalajara	20.0	19.7	20.3	0.93 (0.60, 1.43)
Monterrey	20.0	20.5	19.6	1.00 (0.65, 1.54)
Puebla	20.0	20.2	19.8	0.98 (0.63, 1.51)
Tijuana	20.0	19.2	20.7	0.88 (0.57, 1.37)
**SES**				
D (Lowest)	21.5	19.4	23.3	1 (Ref)
D+	22.4	21.0	23.8	1.06 (0.70, 1.60)
C-	18.7	16.9	20.3	1.00 (0.65, 1.55)
C	24.1	29.9	18.9	1.90** (1.26, 2.87)
C+ (Highest)	13.3	12.8	13.8	1.11 (0.69, 1.80)
**Marital Status**				
Single	10.1	10.3	10.0	1 (Ref)
Married	57.1	55.2	59.1	1.10 (0.69, 1.76)
Cohabitation (*Unión libre*)	17.1	17.7	16.4	0.95 (0.55, 1.64)
Divorced	5.1	5.6	4.6	0.85 (0.40, 1.79)
Widow	2.3	2.6	2.0	0.82 (0.30, 2.25)
Separated	8.3	8.6	7.9	0.95 (0.50, 1.80)
**Age of Youngest Adolescent Child**	14.3 (2.1)	14.2 (2.1)	14.4 (2.1)	0.96 (0.90, 1.03)
**Number of Adolescent Children**				
One	72.9	71.9	73.9	1 (Ref)
Two	23.7	24.3	23.1	1.08 (0.78, 1.50)
Three or more	3.4	3.8	3.0	1.30 (0.61, 2.78)
**Gender of Adolescent Children**				
Males only	51.0	52.2	49.9	1 (Ref)
Females only	34.8	33.8	35.7	0.91 (0.67, 1.22)
Both	14.3	14.1	14.5	0.93 (0.62, 1.40)

*Unión libre* is a relationship status in which couples cohabitate and are romantically involved but their relationship is not recognized by church or law

**p ≤ .01

Nearly half the parents (47.7%) were viewers of *OTF,* watching on average four episodes per week (median was also four episodes). Viewers and non-viewers were comparable across demographics with the exception of respondents in the middle C class where the odds of viewing *OTF* were nearly twice (OR = 1.90; 95% CI = 1.26, 2.87; *p* = .002) compared to parents in the lower D class (see Table 1). Among parents who viewed *OTF*, 25.3% reported co-viewing the *telenovela* with daughters or sons. Fathers co-viewed with sons more than daughters (21.9% vs. 17.3%), and mothers co-viewed with daughters more than sons (33.3% vs. 28.7%).

The bivariate analyses suggest that parent non-viewers, compared to viewers, had more positive attitudes towards abstinence (8.95 vs. 8.44; *t* (818) = 2.00; *p* = .045) and more negative attitudes towards contraception and talking to adolescents about sex (6.59 vs. 5.80; *t* (818) = 3.25; *p* = .001). Co-viewing *OTF* with children was not significantly related to parent-adolescent communication in the last 3 months about SRH and unhealthy romantic relationships, with the exception of mother-daughter discussions of abstinence (*p* = .032; see Additional file 1) – a greater percentage (67.7%) of mothers who co-viewed with daughters discussed abstinence with an adolescent child compared to those who did not co-view with daughters (51.5%).

The multivariable logistic regression models show a significant association between *OTF* viewing and four out of five key outcomes after controlling for demographics and other potential confounders (see Additional file 2). Viewers had significantly higher odds for talking with their adolescent child about sexual relations (OR = 1.69; 95% CI = 1.26, 2.25; *p* < .001), contraceptive methods (OR = 1.46; 95% CI = 1.10, 1.95; *p* = .01), condoms (OR = 1.57; 95% CI = 1.17, 2.09; *p* = .002), and abstinence (OR = 1.57; 95% CI = 1.18, 2.10; *p* = .002) in the last 3 months compared to non-viewers. However, the odds of viewers talking to their adolescent child about unhealthy romantic relationships were not significantly higher than that of non-viewers.

### Adolescents

For the adolescent sample (*n* = 1640), 50% were female, the average age was 16.2 years, and there was an even distribution by residential zone in Mexico. SES ranged from D (lower) to C+ (higher) with a plurality in the middle C class (24.6%). A majority (62.7%) reported no sexual onset (not having had vaginal sex); among those with sexual onset, 45.3% reported having had vaginal sex one to three times in the last 3 months, and 35.1% reported having had sex more often (see Table 2).

**Table 2:** Demographic characteristics of adolescent sample by viewership of *Overcome the Fear*

	Overall (*n* = 1640)	Viewer (*n* = 786)	Non-viewer (*n* = 854)	Bivariate estimate of viewership
	% *n* or *M* (SD)			OR (95% CI)
**Age**	16.2 (2.2)	16.2 (2.2)	16.2 (2.2)	1.00 (0.96, 1.05)
**Gender**				
Male	50.0	49.6	50.4	1 (Ref)
Female	50.0	50.4	49.6	1.03 (0.85, 1.25)
**Metro Zone**				
Mexico Valley	20.0	20.4	19.7	1 (Ref)
Guadalajara	20.0	19.6	20.4	0.93 (0.68, 1.26)
Monterrey	20.0	20.4	19.7	1.00 (0.74, 1.36)
Puebla	20.0	20.2	19.8	0.99 (0.73, 1.34)
Tijuana	20.0	19.5	20.5	0.92 (0.68, 1.25)
**SES**				
D (Lowest)	23.9	22.5	25.2	1 (Ref)
D+	20.0	17.9	21.9	0.92 (0.68, 1.23)
C-	17.9	17.4	18.3	1.07 (0.79, 1.45)
C	24.6	29.3	20.4	1.61*** (1.21, 2.12)
C+ (Highest)	13.6	12.8	14.3	1.01 (0.73, 1.40)
**Sex in last 3 months**				
Has never had sex	62.7	63.5	61.9	1 (Ref)
None	7.3	6.9	7.7	0.87 (0.59, 1.27)
1–3 times	16.9	16.7	17.1	0.95 (0.73, 1.24)
4–6 times	6.4	7.1	5.5	1.26 (0.84, 1.90)
7 or more times	6.8	5.9	7.7	0.74 (0.50, 1.10)

***p ≤ .001

Nearly half (47.9%) were viewers of *OTF,* watching on average four episodes per week (median was also four episodes). Viewers and non-viewers were comparable across demographics with the exception of respondents in the middle C class where the odds of viewing *OTF* were greater (OR = 1.61; 95% CI = 1.21, 2.12; *p* = .001) than adolescents in the lower D class. Among adolescents who viewed *OTF*, 57.6% reported co-viewing the *telenovela* with their mother, while only 9.4% reported co-viewing with their father. Distributions were similar between male and female adolescents – 10% vs. 8.8%, respectively, viewed with fathers, and 58.2% vs. 57.1% viewed with mothers.

The bivariate analyses suggest that adolescent non-viewers, compared to *OTF* viewers, had more negative attitudes towards contraception use (14.65 vs. 13.36; *t*(1638) = 3.71; *p* < .001) and more positive attitudes towards premarital sex (12.26 vs. 11.51; *t*(1638) = 3.09; *p* = .002). Co-viewing *OTF* with a parent was significantly related to contraceptive practices in the last 3 months, with differences present by parent and adolescent gender (see Additional file 1). Male adolescents who co-viewed with a father were more likely than male adolescents who did not co-view with a father to look for contraception information (38.5% vs. 23.4%; *p* = .038), and those who co-viewed with a mother were more likely than those who did not to look for information on unhealthy romantic relationships (12.3% vs. 6.1%; *p* = .042). Among female adolescents, those who co-viewed with a father were more likely than those who did not to look for information on unhealthy relationships (31.4% vs. 10.8%; *p* = .000), and those who co-viewed with a mother were more likely than those who did not to report condom use at last sex (95.3% vs. 73.4%; *p* = .001).

The logistic regression models show a significant association between *OTF* viewing and four key outcomes after controlling for demographics and other potential confounders (see Additional file 3). Viewers had significantly higher odds for seeking information about contraception (OR = 1.67; 95% CI = 1.32, 2.14; *p* < .001) and about unhealthy relationships (OR = 1.56; 95% CI = 1.09, 2.17; *p* = .019), using a contraceptive method other than condoms (OR = 1.75; 95% CI = 1.07, 2.87; *p* = .027), and using dual contraception (OR = 1.77; 95% CI = 1.02, 3.05; *p* = .042) in the last 3 months compared to non-viewers. However, the odds of viewers using a condom at last sex were not significantly higher than that of non-viewers.

## Discussion

Our study suggests that viewership of an EE *telenovela* of high production quality and informed by extensive formative research is related to adolescent health outcomes and parent-adolescent SRH communication on a countrywide scale in Mexico. Despite a highly saturated television landscape in Mexico [40], the *telenovela* reached 3.5 million viewers nightly for over 45 episodes. In addition, its transmedia elements engaged a substantial number of individuals, including over 20 million video views and over 40 million individuals reached on social media. This level of reach is uncommon for a public health intervention and speaks to the enormous potential of an EE television series as an intervention strategy.

Among 12–19-year-olds in Mexico, watching two or more episodes of *OTF* per week was positively related to seeking information about contraception and unhealthy romantic relationships, using another contraceptive method besides condoms, and using dual contraception in the last 3 months. Adolescent viewers were also less likely than non-viewers to report negative attitudes toward contraception.

Adolescent viewers in this study were nearly 80% more likely than non-viewers to recently use dual contraception. Dual contraception is considered ideal for adolescents [41] as it protects against both STIs and pregnancy; however, its use is generally low, and few interventions have proven successful at increasing use [42]. *OTF* included two storylines about dual contraception (one detailed in “Methods”), which could have contributed to this decision among adolescents. Such results are promising and may indicate that *telenovela* storylines can impact SRH behaviors. *OTF* viewership was not related to condom use, however. This may be due to a ceiling effect, as condom use was already fairly high among the sample in general (80% of adolescents who were sexually active in the past 3 months reported using condoms). Importantly, adolescent viewers were also more likely to use a contraceptive method other than condoms.

Among parents of 12–19-year-old adolescents, watching two or more episodes of *OTF* per week was positively related to talking with their adolescent child about sexual relations, contraceptive methods, condoms, and abstinence in the last 3 months. Further, parent viewers were less likely than non-viewers to report positive attitudes toward abstinence and negative attitudes toward contraception and talking to adolescents about sex. *OTF* included storylines about parent-adolescent communication around sexual relations and contraception (detailed in “Methods”), which could have encouraged these behaviors and beliefs among viewers. Some studies have indicated that parent-adolescent discussions about sexual behaviors can encourage contraception use at first sex [18], suggesting that discussions at a younger age or before sexual initiation can impact behavior. However, youth in Mexico are more likely to report parent-adolescent SRH communication if they are sexually active compared to those not yet sexually active [35]. EE *telenovela* interventions like *OTF* can encourage parents to begin discussions about sexual health with their adolescent children, whether they have begun having sex or not.

Among viewers of *OTF*, parent-adolescent co-viewing was significantly related to certain outcomes. Reports of co-viewing were higher among adolescents than parents, in particular with mothers compared to fathers. Parent-adolescent co-viewing has been associated with discussions about the television content [24] and SRH communication [25]. Though parent-adolescent discussions about *OTF* were not measured in this study, it is possible that co-viewing prompted discussions about SRH topics and motivated youth to look for information or use condoms during sex. Similarly, other studies suggest that parent-adolescent discussions about sexual behaviors are more likely to lead to condom use at first sex [18] and that parent-adolescent discussions about SRH tend to be more common with mothers [18, 36]. However, this study suggests that co-viewing an EE *telenovela* with fathers has the potential to encourage adolescents to look for information on contraception and unhealthy romantic relationships.

This study has several unique strengths. To our knowledge, there is only one other *telenovela*-based EE intervention evaluation that examines outcomes among Mexican adolescent and adult viewers [43]; however, this intervention was U.S.-based while our intervention was based in Mexico. Another strength of this study is that it was conducted in a real-world setting, measuring behavioral outcomes of actual viewers. Respondents began and continued watching *OTF* on their own accord, suggesting a lure to the show and to its characters and storylines. The significant associations between viewership and outcomes would suggest that *OTF* might impact behaviors regardless of length of viewership.

Some advantages of partnering with Grupo Televisa to co-produce *OTF* are noteworthy. Grupo Televisa holds a majority of the broadcast television market share in Mexico and was able to mobilize a large promotional campaign for *OTF* before the premiere air date to maximize the *telenovela*’s viewership. Grupo Televisa also guaranteed a broad reach of *OTF* beyond Mexico with a distribution of the *telenovela* to multiple networks across Caribbean and Latin American countries that included Brazil, Chile, Colombia, Costa Rica, El Salvador, Honduras, Nicaragua, Panama, and the Dominican Republic, as well as Canada in North America and *Univision* for the U.S. Hispanic television market. (An evaluation of *OTF* was conducted in the U.S. and will be reported elsewhere). Moreover, the commercial ratings success of *OTF* compelled Grupo Televisa to produce spin-offs that included *Overcome the Heartbreak (Vencer el Desamor*), *Overcome the Past (Vencer el Pasado*), and *Overcome the Absence (Vencer la Ausencia*), where a similar approach was used to develop transitional characters that model healthy behaviors.

This study has several limitations that should be considered. The surveys were cross-sectional, which inhibits our ability to infer a causal relationship between intended outcomes and exposure to *OTF*. However, the positive viewership results on SRH-related topics in both the parent and adolescent samples strengthen the findings. Also, we did not purposely recruit parents and adolescents from the same family, so we were not able to perform dyadic data analyses. However, our approach may have minimized social desirability bias among adolescents, who might have been hesitant about discussing their sexual behaviors in a study that also involved a parent. We assessed *OTF* viewership by asking participants to report the average number of episodes they watched per week, if any. However, we did not ask additional questions to verify viewership. Further, the survey did not measure sexual identity or same-sex sexual behaviors among adolescent respondents, thus we were not able to assess the impact of *OTF* on these adolescents specifically. Finally, the results may not be generalizable to all parts of Mexico. While we conducted surveys in five major metropolitan zones, rural participants may not be represented.

## Conclusions

EE remains an underutilized public health strategy, despite its promise to engage viewers and motivate healthful behaviors on a population scale. The results from this study indicate that a carefully planned EE *telenovela* intervention can have an impact on both adolescent SRH behaviors and parent communication with adolescent children about SRH topics in Mexico. Additional EE *telenovelas* in Mexico, including those already in the works, have the potential to improve SRH adolescent outcomes and reduce rates of unplanned pregnancy across the country.

## Supplementary Information

**Additional file 1 **Percentage of Male and Female Respondents Who Performed Outcomes, by Parent-Adolescent *Overcome the Fear* Co-Viewing.

**Additional file 2.** Multivariable Models Predicting Parental Communication in Last Three Months with Adolescent Child About SRH Topics.

**Additional file 3.** Multivariable Models Predicting Information Seeking and Contraceptive Practices in Last Three Months Among Adolescents.

## Data Availability

Fully de-identified data can be made available from the corresponding author on reasonable request (jorge@sentientresearch.net).

## Abbreviations

EE: Entertainment-education
SRH: Sexual and reproductive health
OTF: Overcome the Fear

## References

[1] Geografia INdEy. Porcentaje de nacimientos registrados de madres adolescentes (menores de 20 años) por entidad federativa de residencia habitual de la madre, serie anual de 2010 a 2020. 2020.

[2] Shamah-Levy TV-OE, Heredia-Hernández O, Romero-Martínez M, Mojica-Cuevas J, Cuevas-Nasu L, Santaella-Castell JA, Rivera-Dommarco J (2020). Encuesta Nacional de Salud y Nutrición 2018–19: Resultados Nacionales.

[3] Orozco-Olvera V, Shen F, Cluver L (2019). The effectiveness of using entertainment education narratives to promote safer sexual behaviors of youth: a meta-analysis, 1985-2017. PLoS One.

[4] Saucier CJ, Suresh S, Brooks JJ, Walter N, Plant A, Montoya JA. The effect of an entertainment-education intervention on reproductive Health of young women of color. Health Commun. 2022;37(9):1093-103. 10.1080/10410236.2021.1903741 33784898

[5] Shelus V, VanEnk L, Giuffrida M, Jansen S, Connolly S, Mukabatsinda M (2018). Understanding your body matters: effects of an entertainment-education serial radio drama on fertility awareness in Rwanda. J Health Commun.

[6] Singhal A (2004). Entertainment-education and social change : history, research, and practice.

[7] Jones SG, Jones R, Fenkl EA, Lacroix-Williams L, Simon S, Chadwell K (2021). Acceptability of the “love, sex, & choices” HIV prevention intervention by Hispanic female college students. Hisp Health Care Int.

[8] Kearney MS, Levine PB (2015). Media influences on social outcomes: the impact of MTV's 16 and pregnant on teen childbearing. Am Econ Rev.

[9] Wang H, Singhal A (2016). East Los high: transmedia edutainment to promote the sexual and reproductive Health of young Latina/o Americans. Am J Public Health.

[10] Frank LB, Falzone P, The Impact of Social Change Communication (2021). Lessons learned from decades of media outreach. Entertainment-education behind the scenes.

[11] Singhal ARE (2001). The entertainment-education strategy in communication campaigns. Public communication campaigns.

[12] Brown WJ, Obregon RWS (2012). Promoting health through entertainment-education media. The handbook of Global Health communication.

[13] Atienzo EE, Campero L, Estrada F, Rouse C, Walker D (2011). Interventions involving parents in order to impact adolescent sexual behavior. Salud Publica Mex.

[14] Coakley TM, Randolph S, Shears J, Beamon ER, Collins P, Sides T (2017). Parent-youth communication to reduce at-risk sexual behavior: a systematic literature review. J Hum Behav Soc Environ.

[15] Gavin LE, Williams JR, Rivera MI, Lachance CR (2015). Programs to strengthen parent-Adolescent communication about reproductive Health: a systematic review. Am J Prev Med.

[16] Wight D, Fullerton D (2013). A review of interventions with parents to promote the sexual health of their children. J Adolesc Health.

[17] Rouvier MCL, Walker D, Caballero M (2011). Factors that influence communication about sexuality between parents and adolescents in the cultural context of Mexican families. Sex Educ.

[18] Atienzo EE, Walker DM, Campero L, Lamadrid-Figueroa H, Gutierrez JP (2009). Parent-adolescent communication about sex in Morelos, Mexico: does it impact sexual behaviour?. Eur J Contracept Reprod Health Care.

[19] Enríquez D (2014). Papel del contexto familiar en la conducta sexual protegida de jóvenes universitarios: Una perspectiva sistémica.

[20] Stern CRC, Fabiola Pérez Baleón GLAM (2020). La comunicación entre familiares y mujeres adolescentes sobre salud sexual y reproductiva como factor preventivo de embarazos en la adolescencia. Los claroscuros del embarazo en la adolescencia: Un enfoque cuantitativo.

[21] Suárez-López LHC, Cruz-Jiménez L, Campero L, Fabiola Pérez Baleón GLAM (2020). Padres, personal docente y profesionales de la salud como fuentes de información sobre salud sexual y reproductiva y su asociación con el embarazo en la adolescencia. Los claroscuros del embarazo en la adolescencia: Un enfoque cuantitativo.

[22] Dorr AKP, Doubleday C (1989). Parent-child coviewing of television. J Broadcast Electron Media.

[23] Padilla-Walker LMCS, Fraser AM (2012). Getting a high-speed family connection: associations between family media use and family connection. Fam Relat.

[24] Bersamin M, Todd M, Fisher DA, Hill DL, Grube JW, Walker S (2008). Parenting practices and adolescent sexual behavior: a longitudinal study. J Marriage Fam.

[25] Dessie Y, Berhane Y, Worku A (2015). Parent-Adolescent sexual and reproductive Health communication is very limited and associated with Adolescent poor behavioral beliefs and subjective norms: evidence from a community based cross-sectional study in eastern Ethiopia. PLoS One.

[26] Guo WNA (2011). The effects of parental mediation of sexual content on the sexual knowledge, attitudes, and behaviors of adolescents in the US. J Child Media.

[27] Fisher DA, Hill DL, Grube JW, Bersamin MM, Walker S, Gruber EL (2009). Televised sexual content and parental mediation: influences on adolescent sexuality. Media Psychol.

[28] Bandura A (1977). Social learning theory.

[29] Bandura A (1986). Social foundations of thought and action : a social cognitive theory.

[30] Slater MDRD (2002). Entertainment—education and elaboration likelihood: understanding the processing of narrative persuasion. Commun Theory.

[31] Green MC, Brock TC (2000). The role of transportation in the persuasiveness of public narratives. J Pers Soc Psychol.

[32] Geografia INdEy. Encuesta Nacional de la Dinámica Demográfica ENADID 2014: Principales resultados 2015.

[33] Geografia INdEy. Natalidad.; 2016.

[34] Lopez-Del Burgo COA, Carlos S, Laris R, Tarasco M, de Irala J (2016). Influence of parent-adolescent relationship on early sexual debut and number of partners among Mexican youth. Medicina y Etica.

[35] Davila SPE, Champion JD, Monsivais MGM, Tovar M, Arias MLF (2017). Mexican Adolescents’ self-reports of parental monitoring and sexual communication for prevention of sexual risk behavior. J Pediatr Nurs.

[36] Gallegos EC, Villarruel AM, Gomez MV, Onofre DJ, Zhou Y (2007). Research brief: sexual communication and knowledge among Mexican parents and their adolescent children. J Assoc Nurses AIDS Care.

[37] United Nations Population Division. World Urbanization Prospects. 2018 Revision. https://population.un.org/wup/Country-Profiles/. Accessed 5 Jan 2021.

[38] Opinión AnMdAdIdMy. Comité de Nivel Socio Económico AMAI, 2018. 2017.

[39] Tavakol M, Dennick R (2011). Making sense of Cronbach's alpha. Int J Med Educ.

[40] Ramírez M. Media Landscapes. European Journalism Centre. 2022. https://medialandscapes.org/country/mexico/traditional-communication/overview. Accessed 13 Feb 2022.

[41] Committee on Adolescent Health C (2017). Committee opinion no 699: Adolescent pregnancy, contraception, and sexual activity. Obstet Gynecol.

[42] Lopez LM, Stockton LL, Chen M, Steiner MJ, Gallo MF. Behavioral interventions for improving dual-method contraceptive use. Cochrane Database Syst Rev. 2014;30(3):CD010915. 10.1002/14651858.CD010915.pub2 PMC10590623 24683022

[43] Lalonde BRP, Shefsky ML, Washienko K (1997). La Esperanza del Valle: alcohol prevention novelas for Hispanic youth and their families. Health Educ Behav.

