# Supplementary material for: Overcome the Fear (Vencer el Miedo): using entertainment education to impact adolescent sexual and reproductive health and parent-child communication in Mexico
Source: BMC Public Health. 2022 Dec 16;22:2366. doi: 10.1186/s12889-022-14853-8 (PMC9757626; doi:10.1186/s12889-022-14853-8)
Supplement: Supplementary file 1 — Additional file 1 Percentage of Male and Female Respondents Who Performed Outcomes, by Parent-Adolescent Overcome the Fear Co-Viewing. [file 12889_2022_14853_MOESM1_ESM.docx]

Additional File 1. Percentage of Male and Female Respondents Who Performed Outcomes, by Parent-Adolescent *Overcome the Fear* Co-Viewing

| In the last three months... | | | Sought information on contraception | | | | Sought information on unhealthy relationships | | |  | Condom use at last sex ^b^ | | Used other contraception (not condoms) | | Used dual contraception | |
| --- | --- | --- | --- | --- | --- | --- | --- | --- | --- | --- | --- | --- | --- | --- | --- | --- |
|  |  | *n* | % *n* | | | *p* | % *n* | | *p* | *n* ^a^ | % *n* | *p* | % *n* | *p* | % *n* | *p* |
| **Female Adolescents** |  |  |  | | |  |  | |  |  |  |  |  |  |  |  |
| Co-viewed: Mother | Yes | 226 | 33.6 | | | .161 | 15.0 | | .095 | 64 | 95.3 | .001 | 21.9 | .165 | 18.1 | .211 |
|  | No | 170 | 27.1 | | |  | 9.4 | |  | 64 | 73.4 |  | 32.8 |  | 28.1 |  |
| Co-viewed: Father | Yes | 35 | 31.4 | | | .934 | 31.4 | | .000 | 5 | 80.0 | .783 | 20.0 | .707 | 20.0 | .853 |
|  | No | 361 | 30.7 | |  | | 10.8 | |  | 123 | 84.6 |  | 27.6 |  | 23.6 |  |
| **Male Adolescents** |  |  |  | |  | |  | |  |  |  |  |  |  |  |  |
| Co-viewed: Mother | Yes | 227 | 26.7 | | .281 | | 12.3 | | .042 | 90 | 81.1 | .994 | 13.3 | .743 | 11.1 | .241 |
|  | No | 163 | 22.1 | |  | | 6.1 | |  | 69 | 81.2 |  | 11.6 |  | 5.8 |  |
| Co-viewed: Father | Yes | 39 | 38.5 | | .038 | | 15.4 | | .211 | 21 | 81.0 | .982 | 9.5 | .650 | 9.5 | .901 |
|  | No | 351 | 23.4 | |  | | 9.1 | |  | 138 | 81.2 |  | 13.0 |  | 8.7 |  |
| Spoke with adolescent child in last three months about... | | | Sexual relations | | | | Contraceptive methods | | |  | Condoms | | Abstinence | | Healthy vs. unhealthy relationships | |
|  |  | *n* | % *n* | *p* | | | % *n* | *p* | | --- | % *n* | *p* | % *n* | *p* | % *n* | *p* |
| **Mothers** |  |  |  |  | | |  |  | |  |  |  |  |  |  |  |
| Co-viewed: Daughters | Yes | 65 | 75.4 | .104 | | | 72.3 | .166 | | --- | 66.2 | .914 | 67.7 | .032 | 72.3 | .237 |
|  | No | 130 | 63.8 |  | | | 62.3 |  | | --- | 66.9 |  | 51.5 |  | 63.8 |  |
| Co-viewed: Sons | Yes | 56 | 64.3 | .519 | | | 67.9 | .679 | | --- | 73.2 | .218 | 60.7 | .497 | 67.9 | .823 |
|  | No | 139 | 69.1 |  | | | 64.7 |  | | --- | 64.0 |  | 55.4 |  | 66.2 |  |
| **Fathers** |  |  |  |  | | |  |  | |  |  |  |  |  |  |  |
| Co-viewed: Daughters | Yes | 34 | 61.8 | .549 | | | 47.1 | .402 | | --- | 50.0 | .391 | 55.9 | .138 | 61.8 | .355 |
|  | No | 162 | 56.2 |  | | | 54.9 |  | | --- | 58.0 |  | 42.0 |  | 53.1 |  |
| Co-viewed: Sons | Yes | 43 | 55.8 | .842 | | | 55.8 | .739 | | --- | 53.5 | .638 | 44.2 | .976 | 51.2 | .609 |
|  | No | 153 | 57.5 |  | | | 52.9 |  | | --- | 57.5 |  | 44.4 |  | 55.6 |  |

*Notes*. Chi-square tests conducted. Analyses include only *OTF* viewers: *n* = 786 adolescents; *n* = 391 parents.

^a^ Among those with sexual onset (*n* = 287).

^b^ Timeframe “in last three months” not applicable to “condom use at last sex.”
