# Supplementary material for: Overcome the Fear (Vencer el Miedo): using entertainment education to impact adolescent sexual and reproductive health and parent-child communication in Mexico
Source: BMC Public Health. 2022 Dec 16;22:2366. doi: 10.1186/s12889-022-14853-8 (PMC9757626; doi:10.1186/s12889-022-14853-8)
Supplement: Supplementary file 2 — Additional file 2. Multivariable Models Predicting Parental Communication in Last Three Months with Adolescent Child About SRH Topics. [file 12889_2022_14853_MOESM2_ESM.docx]

Additional File 2. Multivariable Models Predicting Parental Communication in Last Three Months with Adolescent Child About SRH Topics

|  | Sexual Relations | Contraceptive Methods | Condoms | Abstinence | Healthy vs. Unhealthy Relationships |
| --- | --- | --- | --- | --- | --- |
|  | OR (95% CI) | OR (95% CI) | OR (95% CI) | OR (95% CI) | OR (95% CI) |
| ***OTF* Viewing** |  |  |  |  |  |
| Non-viewer | 1 (Ref) | 1 (Ref) | 1 (Ref) | 1 (Ref) | 1 (Ref) |
| Viewer | 1.69*** (1.26, 2.25) | 1.46** (1.10, 1.95) | 1.57** (1.17, 2.09) | 1.57** (1.18, 2.10) | 1.27 (0.94, 1.70) |
| **Parent Age** | 0.99 (0.96, 1.02) | 0.99 (0.98, 1.02) | 1.00 (0.97, 1.03) | 1.01 (0.98, 1.04) | 1.02 (0.99, 1.05) |
| **Parent Gender** |  |  |  |  |  |
| Father | 1 (Ref) | 1 (Ref) | 1 (Ref) | 1 (Ref) | 1 (Ref) |
| Mother | 1.22 (0.91, 1.63) | 1.20 (0.90, 1.61) | 1.13 (0.84, 1.52) | 1.76*** (1.31, 2.36) | 1.66*** (1.23, 2.25) |
| **Metro Zone** |  |  |  |  |  |
| Mexico Valley | 1 (Ref) | 1 (Ref) | 1 (Ref) | 1 (Ref) | 1 (Ref) |
| Guadalajara | 0.57* (0.36, 0.91) | 0.39*** (0.24, 0.62) | 0.55* (0.35, 0.88) | 0.84 (0.53, 1.31) | 0.89 (0.55, 1.43) |
| Monterrey | 0.54* (0.34, 0.87) | 0.45*** (0.28, 0.73) | 0.59* (0.37, 0.95) | 0.57** (0.35, 0.91) | 0.42*** (0.25, 0.68) |
| Puebla | 0.51** (0.32, 0.81) | 0.42*** (0.26, 0.68) | 0.53** (0.33, 0.84) | 0.67 (0.43, 1.06) | 0.69 (0.43, 1.11) |
| Tijuana | 0.52** (0.33, 0.83) | 0.45*** (0.28, 0.72) | 0.55* (0.35, 0.88) | 0.75 (0.48, 1.06) | 0.54** (0.34, 0.87) |
| **SES** |  |  |  |  |  |
| D (Lowest) | 1 (Ref) | 1 (Ref) | 1 (Ref) | 1 (Ref) | 1 (Ref) |
| D+ | 1.48 (0.96, 2.28) | 1.41 (0.91, 2.18) | 1.36 (0.82, 2.26) | 1.34 (0.86, 2.09) | 1.65* (1.06, 2.56) |
| C- | 1.27 (0.80, 2.00) | 1.42 (0.90, 2.24) | 1.36 (0.89, 2.08) | 1.59* (1.00, 2.53) | 2.04** (1.27, 3.25) |
| C | 1.43 (0.93, 2.18) | 1.24 (0.81, 1.89) | 1.31 (0.83, 2.07) | 1.32 (0.85, 2.03) | 1.75* (1.13, 2.69) |
| C+ (Highest) | 2.07** (1.24, 3.47) | 2.20** (1.31, 3.68) | 1.48 (0.96, 2.29) | 1.84* (1.10, 3.07) | 2.27** (1.35, 3.83) |
| **Marital Status** |  |  |  |  |  |
| Single | 1 (Ref) | 1 (Ref) | 1 (Ref) | 1 (Ref) | 1 (Ref) |
| Married | 0.85 (0.52, 1.41) | 0.71 (0.43, 1.18) | 0.91 (0.55, 1.50) | 1.27 (0.77, 2.11) | 0.81 (0.49, 1.35) |
| Cohabitation (*Unión libre*) | 0.89 (0.50, 1.58) | 0.76 (0.42, 1.35) | 1.15 (0.65, 2.05) | 0.91 (0.51, 1.62) | 1.26 (0.70, 2.28) |
| Divorced | 1.06 (0.48, 2.34) | 0.71 (0.32, 1.57) | 1.34 (0.61, 2.96) | 1.72 (0.78, 3.80) | 0.69 (0.31, 1.54) |
| Widow | 1.18 (0.40, 3.52) | 1.38 (0.45, 4.24) | 1.65 (0.54, 5.05) | 1.18 (0.41, 3.42) | 0.77 (0.26, 2.30) |
| Separated | 1.16 (0.58, 2.29) | 0.81 (0.41, 1.60) | 1.05 (0.54, 2.07) | 1.32 (0.67, 2.59) | 1.44 (0.71, 2.95) |
| **Age of Youngest Adolescent Child** | 1.08* (1.00, 1.17) | 1.07 (0.99, 1.15) | 1.10* (1.02, 1.19) | 1.01 (0.94, 1.10) | 1.02 (0.95, 1.11) |
| **Number of Adolescent Children** |  |  |  |  |  |
| One | 1 (Ref) | 1 (Ref) | 1 (Ref) | 1 (Ref) | 1 (Ref) |
| Two | 1.14 (0.72, 1.80) | 1.34 (0.85, 2.13) | 1.13 (0.72, 1.79) | 1.05 (0.67, 1.68) | 1.53 (0.95, 2.46) |
| Three or more | 3.20* (1.16, 8.78) | 1.98 (0.80, 4.95) | 1.98 (0.78, 5.03) | 2.22 (0.88, 5.59) | 2.67 (0.99, 7.21) |
| **Gender of Adolescent Children** |  |  |  |  |  |
| Males only | 1 (Ref) | 1 (Ref) | 1 (Ref) | 1 (Ref) | 1 (Ref) |
| Females only | 0.81 (0.59, 1.10) | 0.73 (0.53, 1.00) | 0.58*** (0.43, 0.80) | 1.11 (0.81, 1.52) | 1.04 (0.75, 1.43) |
| Both | 0.97 (0.55, 1.73) | 0.93 (0.52, 1.65) | 0.89 (0.51, 1.58) | 1.47 (0.83, 2.58) | 0.84 (0.46, 1.52) |

*Notes*. *N* = 820 for all models. *Unión libre* is a relationship status in which couples cohabitate and are romantically involved but their relationship is not recognized by church or law.

**p* ≤ .05, ***p* ≤.01, ****p* ≤ .001
