# Supplementary material for: Overcome the Fear (Vencer el Miedo): using entertainment education to impact adolescent sexual and reproductive health and parent-child communication in Mexico
Source: BMC Public Health. 2022 Dec 16;22:2366. doi: 10.1186/s12889-022-14853-8 (PMC9757626; doi:10.1186/s12889-022-14853-8)
Supplement: Supplementary file 3 — Additional file 3. Multivariable Models Predicting Information Seeking and Contraceptive Practices in Last Three Months Among Adolescents. [file 12889_2022_14853_MOESM3_ESM.docx]

Additional File 3. Multivariable Models Predicting Information Seeking and Contraceptive Practices in Last Three Months Among Adolescents

|  | Sought information on contraception | Sought information on unhealthy  relationships | Condom use at last sex | Other contraception use (not condoms) | Use of dual contraception |
| --- | --- | --- | --- | --- | --- |
|  | OR (95% CI) | OR (95% CI) | OR (95% CI) | OR (95% CI) | OR (95% CI) |
| ***OTF* Viewing** |  |  |  |  |  |
| Non-Viewer | 1 (Ref) | 1 (Ref) | 1 (Ref) | 1 (Ref) | 1 (Ref) |
| Viewer | 1.67*** (1.32, 2.14) | 1.56* (1.09, 2.17) | 1.17 (0.76, 1.79) | 1.75* (1.07, 2.87) | 1.77* (1.02, 3.05) |
| **Adolescent Age** | 1.04 (0.98, 1.11) | 1.01 (0.92, 1.11) | 1.02 (0.89, 1.17) | 1.23* (1.03, 1.47) | 1.42*** (1.15, 1.77) |
| **Adolescent Gender** |  |  |  |  |  |
| Male | 1 (Ref) | 1 (Ref) | 1 (Ref) | 1 (Ref) | 1 (Ref) |
| Female | 1.42** (1.11, 1.80) | 1.38 (0.98, 1.95) | 1.62* (1.05, 2.50) | 3.62*** (2.19, 6.00) | 4.21*** (2.38, 7.44) |
| **Metro Zone** |  |  |  |  |  |
| Mexico Valley | 1 (Ref) | 1 (Ref) | 1 (Ref) | 1 (Ref) | 1 (Ref) |
| Guadalajara | 0.91 (0.63, 1.32) | 0.66 (0.40, 1.08) | 0.97 (0.49, 1.92) | 0.62 (0.26, 1.48) | 0.55 (0.20, 1.49) |
| Monterrey | 0.42*** (0.27, 0.63) | 0.24*** (0.12, 0.47) | 0.76 (0.38, 1.51) | 0.34* (.14, 0.87) | 0.41 (0.15, 1.12) |
| Puebla | 1.14 (0.80, 1.63) | 0.96 (0.60, 1.53) | 1.65 (0.83, 3.28) | 1.32 (0.64, 2.73) | 1.31 (0.58, 2.93) |
| Tijuana | 0.88 (0.61, 1.27) | 0.52* (0.31, 0.88) | 1.66 (0.83, 3.33) | 0.67 (0.31, 1.44) | 0.87 (0.38, 1.99) |
| **SES** |  |  |  |  |  |
| D (Lowest) | 1 (Ref) | 1 (Ref) | 1 (Ref) | 1 (Ref) | 1 (Ref) |
| D+ | 1.32 (0.91, 1.92) | 1.73 (0.98, 3.06) | 1.92 (0.98, 3.77) | 1.17 (0.54, 2.53) | 1.19 (0.50, 2.83) |
| C- | 1.12 (0.76, 1.66) | 1.88* (1.06, 3.33) | 1.14 (0.59, 2.19) | 0.95 (0.41, 2.19) | 1.29 (0.52, 3.16) |
| C | 1.21 (0.85, 1.72) | 1.55 (0.89, 2.70) | 1.31 (0.71, 2.41) | 1.69 (0.82, 3.49) | 1.92 (0.86, 4.27) |
| C+ (Highest) | 1.73** (1.16, 2.58) | 2.13* (1.17, 3.89) | 1.45 (0.74, 2.84) | 1.54 (0.68, 3.46) | 1.60 (0.65, 3.93) |
| **Sex in last 3 months** |  |  |  |  |  |
| Has never had sex | 1 (Ref) | 1 (Ref) | Not included | Not included | Not included |
| None | 1.16 (0.71, 1.88) | 2.32** (1.30, 4.14) | 1 (Ref) | 1 (Ref) | 1 (Ref) |
| 1-3 times | 1.96*** (1.39, 2.76) | 1.45 (0.87, 2.41) | 1.01 (0.54, 1.88) | 11.45*** (2.67, 49.12) | 8.54** (1.97, 37.12) |
| 4-6 times | 2.31*** (1.43, 3.73) | 1.02 (0.47, 2.24) | 0.70 (0.34, 1.46) | 15.63*** (3.51, 69.68) | 8.93** (1.95, 40.83) |
| 7 or more times | 2.31*** (1.44, 3.71) | 2.38** (1.26, 4.49) | 0.32 (0.17, 0.63) | 20.21*** (4.54, 90.03) | 15.03*** (3.33, 67.76) |

*Notes*. *N* = 1640 for first two models. *N* = 612 for last three models, which include only adolescents who reported sexual onset.

**p* ≤ .05, ***p* ≤.01, ****p* ≤ .001
